# 

**Published:** 1908-07-04

**Authors:** 


### BEQUESTS TO CHARITIES.

The late Dr. Bickersteth has left £10,000 to the Liverpool Royal Infirmary. In accordance with the donor's wishes the money will be devoted to extending and improving the out-patients' department. It is understood that the governors of the infirmary propose to undertake a much larger extension than is provided for by this gift, and that they will make a public appeal.

Mr. Charles Edwin Layton, of Cornwall Terrace, Regent's Park, bequeathed £6,000 to King's College, £2,000 to King's College Hospital, £5,000 to Charing Cross Hospital, £2,000 to the National Hospital for Consumption, Ventnor, £500 to the Seaford Convalescent Hospital, £500 to the Princess Alice Memorial Hospital, Eastbourne, £1,000 to the Metropolitan Sunday Hospital Fund, £300 to the Hospital of St. Luke, and about £34,000 to various non-medical charities. The residue is to be divided in equal shares between the Stationers' Company, the building fund of St. Bartholomew's Hospital, and the Church Pastoral Aid Society. Mr. Layton left an estate of £94,132.

For the convenience of travellers to Belgium by the Harwich route, the Great Eastern Railway Company have just placed on the Antwerp express train from Liverpool Street Station, dining and breakfast cars, in which *table d'hôte* dinners and other refreshments are served on the down journey and *table d'hôte* breakfast on the up journey. The Radiotelegraphic Convention came into force on July 1, and the Great Eastern Railway Company have fitted the turbine steamer *Copenhagen* and the twin-screw *Dresden* with wireless telegraphy, which will enable the public to send telegrams from on board these vessels from now onwards. The other steamers on the Hook of Holland service are also being provided with wireless telegraphy.

### THE GRADUATES' MEDICAL SCHOOLS.

DIARY FOR WEEK JULY 6 TO 10.

### THE HOSPITAL FOR SICK CHILDREN, Great Ormond Street, W.C.

**At 4 p.m.**
**July 9,** Mr. Kellock, **Necrosis of the Long Bones.**

### NATIONAL HOSPITAL FOR THE PARALYSED AND EPILEPTIC, Queen Sq., Bloomsbury, W.C.

**At 3.30 p.m.**
**July 7,** Dr. Beevor, **Action of Muscles.**
**July 10,** Mr. Armour, **Surgery of the Nervous System.**

### LONDON HOSPITAL MEDICAL COLLEGE, Turner Street, Mile End, E.

**At 3 p.m.**
**July 7,** Mr. Jonathan Hutchinson, F.R.S., **The Present State of the Leprosy Question.**

### MOUNT VERNON HOSPITAL, Northwood.

**At 5 p.m.**
**July 9,** Dr. G. F. Johnston, **Demonstration at the Sanatorium.**

### CHARING CROSS HOSPITAL, W.C.

Post Graduate Demonstrations.

**At 4 p.m.**
**July 9,** Dr. Bosanquet, **Medical.**

### THE POST GRADUATE COLLEGE, West London Hospital, Hammersmith, W.

**At 12 noon.**
**July 6,** Dr. Low, **Pathological Demonstration.**
**At 12.15 p.m.**
**July 8** and **10,** Dr. Pritchard, **Practical Medicine.**
**At 5 p.m.**
**July 6,** Mr. Baldwin, **Practical Surgery—V.**
**July 7,** Dr. Low, **Human Trypanosomiasis (Sleeping Sickness).**
**July 8,** Mr. Lloyd Williams, **The Dental Examination of School Children.**
**July 9,** Mr. Keetley, **Clinical—II.**
**July 10,** Mr. R. W. Lloyd, **Anæsthetics—II.**

### MEDICAL GRADUATES' COLLEGE AND POLYCLINIC, Chenies Street, W.C.

**At 5.15 p.m.**
**July 6,** Mr. B. G. A. Moynihan, **Duodenal Ulcer.**
**July 7,** Mr. C. H. Leaf, **Surgical Treatment in so-called Inoperable Cancer.**
**July 8,** Dr. John Turner, **Dementia Præcox.**
**July 9,** Dr. R. H. A. Whitelocke, **The Injuries Incidental to Athletic Exercises.**

### LONDON SCHOOL OF CLINICAL MEDICINE, Seamen's Hospital, Greenwich, S.E.

**At 2.30 p.m.**

**July 2,** Dr. Rankin, **Myxœdema.**

